# Comment on: Kadomoto, S. et al. “Tumor-Associated Macrophages Induce Migration of Renal Cell Carcinoma Cells via Activation of the CCL20-CCR6 Axis” *Cancers* 2020, *12,* 89

**DOI:** 10.3390/cancers12020342

**Published:** 2020-02-03

**Authors:** Karin Zins, Dietmar Abraham

**Affiliations:** Center for Anatomy and Cell Biology, Division of Cell and Developmental Biology, Medical University of Vienna, Vienna 1090, Austria; karin.zins@meduniwien.ac.at

Macrophages form a major component of the leukocyte infiltrate in solid tumors and it has become increasingly clear that tumor-associated macrophages (TAMs) have tumor-promoting effects within the stroma [1]. Renal cell carcinoma (RCC) solid tumors are comprised of a heterogeneous microenvironment of both malignant and normal stromal cells containing large numbers of macrophages [2].

We read with interest the paper by Suguru Kadomoto et al. entitled “Tumor-associated macrophages induce migration of renal cell carcinoma cells via activation of the CCL20-CCR6 axis”, published in *Cancers* [3], in which they report that the CCL20-CCR6 axis induces migration and epithelial–mesenchymal transition (EMT) of ACHN and Caki-1 RCC cells in co-cultures with THP-1/U937-derived tumor conditioned macrophages.

We would like to widen the focus of the paper and make some additional comments. Importantly, we would like to point out the unclear macrophage polarization status of the phorbol 12-myristate 13-acetate (PMA)-treated cells used in the study, which the authors addressed in the discussion. The study is based on co-culture data from PMA-stimulated monocytic THP-1 and U937 cells that result in macrophage-like cells, probably lacking a specific M1 or M2 phenotype. Unfortunately, the resulting macrophage phenotype is insufficiently characterized by the authors thereby limiting the significance of the findings.

In order to improve the informative value of this study, it would be useful to perform an additional M1-polarization of PMA-treated cells by interferon IFN-γ and toll-like receptor (TLR) ligand lipopolysaccharide (LPS) treatment, which leads to the secretion of high amounts of proinflammatory cytokines [4]. In addition, activation of a M2 phenotype by factors such as IL4, IL13, IL10 and colony stimulating factor 1 (CSF1) was not performed. Such treatments should lead to low production of proinflammatory cytokines, high production of the anti-inflammatory cytokines IL10 and cyclooxygenase-2 (COX-2) derived prostaglandin E2 (PGE2) as well as high expression of mannose (CD206) and scavenger (CD163) receptors [4]. Without clear profiling of the potential macrophage phenotypes present in the co-culture setting with a set of M1, M2 and TAM markers, the data do not support the conclusions presented.

Importantly, TAMs do not fit entirely into the criteria for M1 or M2 macrophages [5] and patient data suggest that TAMs in RCC show a mixed M1/M2 phenotype [6]. Of interest, TAMs expressing high amounts of T-cell immunoglobulin and mucin domain-containing molecule-3 (TIM-3), which is not a classical M1 or M2 marker, were associated with poor prognosis in clear cell RCC [7]. Moreover, the specific tumor microenvironment promotes differentiation of macrophages to heterogenous pro-tumoral TAM phenotypes in the same tumor [1]. Thus, we believe that PMA-treated THP-1 and U937 cells poorly reflect the situation in patient tumors. In future studies, the use of patient-derived TAMs from RCC cancer patients would be an alternative approach to address the role of TAMs in RCC in an in vitro setting.

To support their findings, the authors performed immunohistochemical staining of CCR6 and CD68 in 42 RCC tissue samples and reported no association between CCR6 and CD68 positive tissues. CCL20 staining was not performed which would be necessary to identify potential overlap and correlation of TAM and CCL20 tissue distribution. In addition, the data do not address the question of whether TAMs are the major stromal source of CCL20 in RCC patients, which could be examined by isolating cancer cells, macrophages, infiltrating leukocytes and cancer associated fibroblasts from patients followed by measurement of CCL20 secretion in the cultures. Consequently, the co-culture data reported in this study are not supported by patient data. 

It would also be better to stain patient tissues with M1 and M2 markers in addition to the general macrophage marker CD68. In line with this suggestion, a previous study analyzed the presence of M1 and M2 macrophages in 185 RCC patients using histological techniques [6]. Using CD68 as a pan-macrophage marker, CD11c for M1 and CD206 as a M2-marker revealed that CD68 alone has a poor predictive value, whereas low CD11c and high CD206 expression as single variables correlated with reduced survival, whereas patients with high CD11c and low CD206 expression had the best survival prognosis [6]. In this context, it should be noted that CD68 was not needed in this analysis. In light of these findings, it is not surprising that CD68 staining did not result in any additional useful knowledge in the present study and underlines the necessity of a complex analysis, using markers for M1, M2 and TAM phenotypes.

Given the important role of TAMs in the progression of RCC and the reported overexpression of CCR6 by cancer cells and aberrant signaling by its ligand CCL20 in many cancer types including colorectal, pancreatic and melanoma solid tumors [8,9,10,11] as well as the development of anti-cancer therapeutics inhibiting CCR6-CCL20 activity [12], investigation of the involvement and mechanisms of the CCL20-CCR6 axis contributing to the development and progression of renal cell cancer is a worthwhile undertaking.
